# A New Method of Reducing Dislocations of the Elbow Joint Backwards

**Published:** 1849-08

**Authors:** 


*A new method of reducing dislocations of the elbow joint backwards.* By M. Maisonneuve.—A case of dislocation of the elbow joint backwards, of some weeks standing, that had resisted the ordinary means of reduction, readily yielded to the following very rational and ingenious method:

The counter-extension strap, instead of passing under the arm pits, was fixed upon the arm itself, immediately below the insertion of the deltoid, the prominence of which served as the fulcrum.

Extension was performed by fixing the strap directly upon the olecranon, instead of the wrist; the two extremities, brought in front, were crossed upon the anterior face of the forearm, then carried behind and crossed a second time upon the posterior face of this part. The forearm was in this way embraced by a double circle in the form of a figure 8. A few moments of gradual and moderate traction were sufficient to return the displaced joint to its home.—*Gazette de Hôpitaux.*—D.W.Y.

